# Androgen secreting steroid cell tumor of the ovary in a young lactating women with acute onset of severe hyperandrogenism: a case report and review of literature

**DOI:** 10.1186/1752-1947-1-182

**Published:** 2007-12-18

**Authors:** Altaf Gauhar Haji, Shekhar Sharma, Manoj Babu, DK Vijaykumar, K Chitrathara

**Affiliations:** 1 Department of Surgical Oncology, Amrita Institute of Medical Sciences and Research Centre, Edapally, Kochi, Pin-682026, Kerela, India

## Abstract

**Introduction:**

Steroid cell tumors of the ovary account for less than 0.1% of all ovarian tumors [1] and these tumours may present at any age in association with interesting presentations related to the hormonal activity and virilizing properties of tumor. Hayes and Scully [2] reported 63 cases in patients ranging from 2 to 80 years of age. The subtype, not otherwise specified, is associated with androgenic changes in approximately one half of patients with this tumour [1]. In a series of 63 cases from Massachusetts General Hospital, 94% of the tumors were found to be unilateral and 28.6% were malignant [3]. As most of these tumors are diagnosed at an early stage and do not recur or metastasize, little is known about their response to therapies such as chemotherapy or radiation [3].

**Case Presentation:**

We present the case of a 22-year old lactating woman who presented with four months of amenorrhea associated with signs of virilization. Clinical and diagnostic evaluation revealed a right adenexal mass and elevated serum levels of testosterone and she was diagnosed as having a stage 1A androgen secreting steroid cell tumor. In view of the early stage of the disease, she underwent right salpingo-oopherectomy. Histopathological examination and immunohistochemistry confirmed the diagnosis. Two months after surgery she regained normal menses and showed regression of the androgenic changes.

**Conclusion:**

Surgery remains the mainstay of the treatment of gonadotrophin receptor positive steroid cell tumors although medical therapy using Gonadotrophin Releasing Hormone [GnRH analogues has been tried recently in recurrent or inoperable cases. There is no described effective chemotherapy or radiotherapy for this condition.

## Introduction

Steroid cell tumors of the ovary account for less than 0.1% of all ovarian tumors [1] and these tumors may present at any age in association with interesting presentations related to the hormonal activity and virilizing properties of tumor. Hayes and Scully [2] reported 63 cases in patients ranging from 2 to 80 years of age. The subtype, not otherwise specified, is associated with androgenic changes in approximately one half of patients with this tumor [1]. In a series of 63 cases from Massachusetts General Hospital, 94% of the tumors were found to be unilateral and 28.6% were malignant [3]. As most of these tumors are diagnosed at an early stage and do not recur or metastasize, little is known about their response to therapies such as chemotherapy or radiation [3].

## Case presentation

This is the case of a 22-year-old uniparous lactating woman who regained regular menses after 8 months of lactational amenorrhea. After a period of six months of regular menstruation she presented with a history of sudden onset of amenorrhea and abnormal hair growth on the face of four months duration. Physical examination revealed a male pattern of coarser hair distribution in the beard region, anterior chest wall and arms. Abdominal examination showed no abnormality and vaginal examination disclosed clitoromegaly. Ultrasonography of the abdomen and pelvis revealed a solid right adenxal mass measuring 51 × 54 × 58 mm with moderate to significant vascularity. There was no ascites, retroperitoneal lymphadenopathy, adrenal gland enlargement, or liver metastasis. Tumor markers and hormonal levels were evaluated [Table 1] and showed raised serum testosterone levels.

**Table 1:** Values of Hormone/tumor marker levels

Marker/Hormone	Test value	Normal values
CA-125	< 4 Units/ml	0–35 Units/ml
β-HCG	0.505 mIU/ml	< 5 mIU/ml
S. Testosterone	309 ngm/ml	0.1–10 ngm/ml (In an adult female)
Alfa Fetoprotein	2.19 ngm/ml	0–15 ngm/ml

The patient underwent an exploratory laparotomy. Intra-operatively the right ovary showed a 5 × 5 cm firm, tan-brown, well-encapsulated mass without any adhesions to surrounding structures and with engorged ovarian vessels. In view of the diagnosis of stage 1A disease, unilateral right salpingo-oopherectomy alone was performed since the frequency of lesions occurring bilaterally in this stage has been reported to be only 6%.

**Figure 1:**
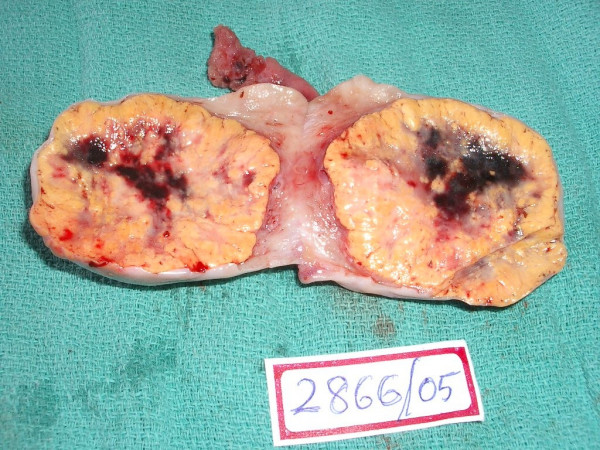
Cut section of the specimen.

**Figure 2:**
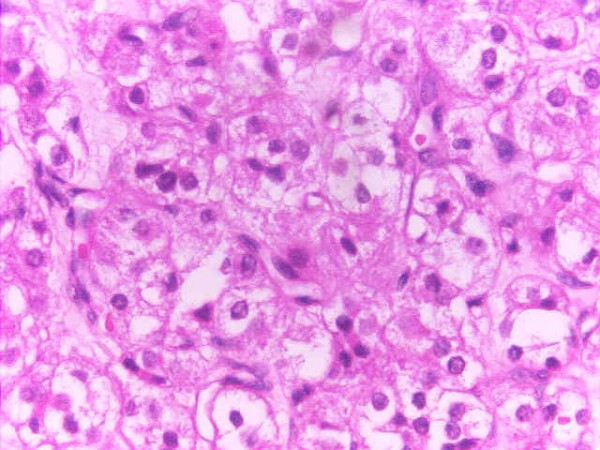
Histopathology.

The cut-section of the specimen showed a tan-brown well-circumscribed tumor with areas of hemorrhage [Figure 1]. Microscopic examination [Figure 2] showed diffuse sheets of large polygonal tumor cells with vacuolated cytoplasm and vesicular nuclei along with nuclear pleomorphism. Crystals of Reinke, which are usually seen in hilus tumors, were not seen.

Immunohistochemistry staining was positive for gonadotrophin receptors and vimentin, and negative for alpha-fetoprotein and epithelial membrane antigen, providing evidence in favour of a steroid cell tumor.

At post operative follow-up, two months of surgery, the patient had regained normal menses. Her serum testosterone level had gone down to 0.262-ngm/ml, and there was regression of the abnormal hair pattern. The patient is being followed up regularly with measurement of hormone levels as marker of recurrence.

## Discussion

Ovarian steroid cell tumors account for less than 0.1% of all ovarian tumors. They are grouped under sex-chord stromal tumors and are usually benign, unilateral and characterized by a steroid cell proliferation [1] &[3].

They are further divided into three subtypes: stromal leuteoma, Leydig cell tumor, and steroid cell tumor [1], [3] &[4], the latter being the most common of the three subtypes accounting for approximately 60% of cases.

Steroid cell tumors are associated with androgenic changes with variable frequency, ranging from 12% to over 50 % [1][4][5] &[6], and they are usually of many years duration[1] &[3]. This case is unique because of its acute presentation, with sudden onset of amenorrhea and signs of virilization, over a four month period, following resumption of menses after a normal delivery.

Historically these tumors have been variously referred to as lipoid cell tumors, lipid cell tumors, adrenal-like tumors, masculinovoblastomas, leutomas, hypernephroid tumors, and adrenal rest tumors, indicating the infidelity of their supposed lineage [1], [2], [3][5]. However, it is postulated that these are of adrenal cortical rest origin, residing near the ovary, on the basis of the presence of mRNA for steroidogenic p450c11 and p450c21, which are expressed only in tissues of adrenal origin [7]. However this theory is still disputed [1].

The majority of steroid cell tumors have a benign or low-grade behavior. Interestingly, pathologically-benign tumors can behave in a clinically malignant fashion [8]. About 20% patients develop metastatic lesions usually within the peritoneal cavity, and rarely at distant sites [2] &[9]. In a series of 63 cases from the Massachusetts General Hospital, 94% of the tumors were found to be unilateral, and 28.6% cases had features suggestive of malignant nature [2].

Hayes and Scully [2] have identified five pathologic features that are highly associated with malignancy: More than two mitoses per high-power field; necrosis; size of 7 cm or larger; hemorrhage; and Grade 2 or 3 nuclear atypia. The final pathologic diagnosis is based on the characteristic histological appearance, including polygonal to round cells with distinct cell borders and an abundant cytoplasm and the absence of Reinke's crystals, and a characteristic pattern of immunohistochemistry staining for inhibin and vimentin [75%], anti human cytokeratin [46%], and AE1/AE3 [37%] [1].

The primary treatment is surgical extirpation of the primary lesion, and there are no reports of effective radiation or chemotherapy [10]. In a young patient with stage IA disease, a unilateral salpingo-oophorectomy is adequate since the frequency of bilateral occurrence is only 6% ( [1], [2] &[10]).

The main reason for poor understanding of the therapeutic value of chemotherapy and radiotherapy in the treatment of these tumors is due to the rarity of this tumor. In recent years attempts have been made to describe the use of Gonadotrophin Releasing Hormone [GnRH] analogues to induce a suppression of secretions and an apoptosis leading to a non-surgical cure [11], [12] &[13]. Pascale, Pugeat and Roberts suggested that androgen secretion by ovarian virilizing tumor is gonadotrophin dependent [11]. They concluded that various ovarian androgen secreting tumors are not autonomous but apparently depend on continuous gonadotrophin stimulation.

Imai A, Lida K, Tamaya T suggested the various relationships between GnRH receptor and tumor growth. Increased Fas ligand level within GnRH receptor-bearing tumors might promote apoptotic cell death through an attack on intra-tumoral Fas positive cells that could, at least in part, account for the anti-proliferative action of the hormone [12]. The coupling of GnRH receptor to the Gi protein subfamily may be a signaling pathway by which GnRH acts in peripheral tumor [12].

So far these approaches have been tried primarily in non-operable cases or recurrent disease. Therefore, at best, these treatments can be considered experimental in the present times. Further insight into the nature, biology and behavior of these tumors may change the gold standard of treatment from surgical extirpation to medical management over time.

## Conclusion

Ovarian steroid cell tumors, grouped under sex-chord stromal tumors, account for less than 0.1% of all ovarian tumors. They are usually benign, unilateral and are characterized by a steroid cell proliferation [1] &[3]. These tumors present with long history of often many years of androgenic changes with variable frequency[1], [3], [4] &[5]. This case is unique because of its acute presentation over a four month period. The primary treatment is surgical extirpation of the primary lesion, and there are no reports of effective treatment with radiation or chemotherapy [10]. In a young patient with stage IA disease, a unilateral salpingo-ophorectomy is adequate since the frequency of bilateralism is only 6% [1], [2] &[10].

## Competing interests

The author(s) declare that they have no competing interests.

## Authors' contributions

AGH contributed in the conceptualization and designing of the manuscript, literature search, data acquisition, data analysis, manuscript preparation, editing, and manuscript review. SS helped in the literature search, data acquisition, data analysis, manuscript preparation, editing, and manuscript review. MB contributed in the literature search, data acquisition, data analysis, manuscript preparation, editing, and manuscript review. DKV contributed in the data analysis, editing, and manuscript review and was the clinician responsible for making the treatment decisions on the patients. KC helped in the editing and manuscript review. All authors read and approved the final manuscript.

## Consent

The patient was informed about the intent to publish this report and consented to the same in writing.
